# Maintenance nifedipine therapy for preterm symptomatic placenta previa: A randomized, multicenter, double-blind, placebo-controlled trial

**DOI:** 10.1371/journal.pone.0173717

**Published:** 2017-03-23

**Authors:** Eric Verspyck, Claire de Vienne, Charles Muszynski, Michael Bubenheim, Isabella Chanavaz-Lacheray, Michel Dreyfus, Philippe Deruelle, Jacques Benichou

**Affiliations:** 1 Department of Obstetrics and Gynecology, Rouen University Hospital, Rouen, France; 2 Department of Obstetrics and Gynecology, Caen University Hospital, Caen, France; 3 Department of Obstetrics and Gynecology, Amiens University Hospital, Amiens, France; 4 Department of Biostatistics, Rouen University Hospital, Rouen, France; 5 Department of Obstetrics and Gynecology, Belvedere Hospital, Mont-Saint-Aignan, France; 6 Department of Obstetrics and Gynecology, Lille University, EA 4489—Environnement Périnatal et Santé, Lille, France; 7 Inserm U657, University of Rouen and Biostatistics, Rouen, France; Public Library of Science, FRANCE

## Abstract

**Objective:**

To assess the impact of maintenance nifedipine therapy on pregnancy duration in women with preterm placenta previa bleeding.

**Methods:**

PPADAL was a randomized, double-blind, placebo-controlled trial conducted between 05/2008 and 05/2012 in five French hospitals. The trial included 109 women, aged ≥ 18 years, with at least one episode of placenta previa bleeding, intact membranes and no other pregnancy complication, at gestational age 24 to 34 weeks and after 48 hours of complete acute tocolysis. Women were randomly allocated to receive either 20 mg of slow-release nifedipine three times daily (n = 54) or placebo (n = 55) until 36 + 6 weeks of gestation. The primary outcome for the trial was length of pregnancy measured in days after enrolment. Main secondary outcomes were rates of recurrent bleeding, cesarean delivery due to hemorrhage, blood transfusion, maternal side effects, gestational age at delivery and adverse perinatal outcomes (perinatal death, chronic lung disease, neonatal sepsis, intraventricular hemorrhage > grade 2, perventricular leukomalacia > grade 1, or necrotizing enterocolitis). Analysis was by intention to treat.

**Results:**

Mean (SD) prolongation of pregnancy was not different between the nifedipine (n = 54) and the placebo (n = 55) group; 42.5 days ± 23.8 versus 44.2 days ± 24.5, p = 0.70. Cesarean due to hemorrhage performed before 37 weeks occurred more frequently in the nifedipine group in comparison with the placebo group (RR, 1.66; 95% confidence interval, 1.05–2.72). Adverse perinatal outcomes were comparable between groups; 3.8% for nifedipine versus 5.5% for placebo (relative risk, 0.52; 95% confidence interval 0.10–2.61). No maternal mortality or perinatal death occurred.

**Conclusion:**

Maintenance oral nifedipine neither prolongs duration of pregnancy nor improves maternal or perinatal outcomes.

**Trial registration:**

ClinicalTrials.gov NCT00620724

## Introduction

A United States population-based study found the overall annual incidence of placenta previa to be 4.8 per 1000 deliveries [1]. Placenta previa rate has regularly increased over the last two decades, as has frequency of the associated main risk factors i.e. cesarean section and maternal age [2]. Placenta previa may have considerable consequences for both the mother and the neonate while placing high demands on health resources. High morbidities and mortality rates reported in women are mainly related to severe hemorrhage occurring throughout pregnancy, though mostly at delivery [3]. Perinatal mortality, the rates of which are three to four times higher than in normal pregnancies, frequently occurs in situation of severe prematurity, mainly induced by maternal bleeding [4]. Since the 1980’s, conservative expectant management has been advocated as reducing both maternal and neonatal morbidity in preterm bleeding placenta previa [5]. Tocolytic agents is one of the key treatments in conservative management and may be useful in selected cases, enabling better control of uterine activity and reducing blood loss [6,7]. The rationale for the selective use of tocolytic agents is twofold. In some women, a significant degree of uterine activity is observed during an episode of bleeding. More often, an episode of bleeding may occur following subclinical uterine contractions [8]. As reported treatment protocols can vary in use of tocolytic agents and administration duration, the Royal College of Obstetricians and Gynecologists have recently stated that tocolytic agent and optimum regime are still to be determined and further research is needed in this area [6]. Prolonged use of tocolytic therapy has until now been poorly investigated in women with bleeding preterm placenta previa [9] with most publications being retrospective and observational and few data reporting maternal morbidity with severe hemorrhage and neonatal morbidity with prematurity [10,11]. A systematic review of randomized controlled trials concluded that calcium channel blockers are preferable to other tocolytic agents (mainly beta-mimetics) in women at risk of preterm delivery [12], but no previous study has described calcium channel blockers therapy in symptomatic preterm bleeding placenta previa. Therefore, we evaluated the effectiveness of maintenance tocolysis with calcium channel blocker nifedipine on pregnancy prolongation in women with preterm symptomatic placenta previa.

## Methods

We performed a multicenter, double blind, placebo-controlled trial in 5 perinatal units including all tertiary centers in the North West of France. Randomization of participants occurred between May 2008 and May 2012 and follow- up was completed in December 2013. The study was approved by the Ethics Committee of Upper Normandy and written informed consent was obtained from each woman. The study was registered with the clinical trials registry and before it recruited the first patient (ClinicalTrials.gov#NCT00620724).

### Participants

Women with preterm placenta previa bleeding, at gestational age 24+0 to 33+6 weeks, and after 48 hours of complete acute tocolysis and corticosteroids were eligible for participation. Placenta previa was diagnosed using transvaginal sonography and a criterion for inclusion was a placental edge below 50 mm from the internal cervical os [7]. Transvaginal and abdominal sonographies were performed before randomization to detect placental abruption and could be further carried out in women with persistent bleeding. Gestational age was determined using the first trimester routine ultrasound examination and by measuring crown-rump length. Acute tocolysis treatment was usually by nifedipine (the initial dose was 10 mg capsules orally renewable every 20 minutes up to 4 capsules per hour in the first hour, followed by 20 mg slow-release tablets three times daily for 48 hours) or atosiban (a bolus injection of 6.75 mg i.v. in 1 minute, followed by 18 mg/hour for 3 hours, followed by a maintenance dosage of 6 mg/hour for 45 hours), following local protocol.

Inclusion criteria were women aged ≥ 18 with a singleton fetus and intact membranes. Women were excluded if any of the following criteria was encountered: severe hemorrhage with immediate delivery required, pre-eclampsia, placental abruption, fetal distress, intrauterine growth restriction, intrauterine fetal death, chorioamnionitis, liver disease, severe chronic renal disease, heart disease, and contra indications for nifedipine.

### Interventions

Women were randomly allocated (the second day of acute tocolysis by a senior obstetrician patrician) to receive either 20 mg of slow-release nifedipine three times daily (60 mg) [12] or placebo until 36 + 6 weeks of gestation. The trial medication commenced after initial acute tocolysis had been discontinued. Side effects related to nifedipine therapy i.e. symptomatic hypotension, headache or flushing were recorded. Antenatal corticosteroid regimen i.e. intramuscular betamethasone (Celestone) 12 mg every 24 hours for two days was systematically given after 26 weeks of gestation and a repeat dose could be re-injected in cases of bleeding recurrence or pregnancy complications. The anesthesiologist and obstetrician jointly decided upon maternal transfusion depending on the mother's hemodynamic situation, the extent of blood loss and following French guidelines [13]. Iron tablets i.e. oral 80 mg daily, were systematically prescribed. Women were kept under observation in hospital for a few days and authorized for discharge if the clinical situation was stable with no persistent bleeding [14]. Women were given instructions to limit activity and to return immediately to hospital should bleeding recur. Women presenting at the hospital with bleeding recurrence could have the study medication stopped for a repeat course of 48 hour acute tocolysis i.e. nifedipine, atosiban or twice consecutively if necessary. A repeat course of acute tocolysis was indicated in women with moderate bleeding and stable hemodynamic situation and when bleeding recurrence occurred before 35 weeks of gestation. After two bleeding recurrences, women were definitively hospitalized. After the initial hospital discharge patients underwent monthly clinical examination including blood pressure measurement, tolerance for study treatment, evaluation for medication compliance by counting pills, and localization of the low placental edge. Trial medication could be further stopped in women with severe hemorrhage, premature rupture of membranes or pregnancy complications (preeclampsia, intra uterine growth retardation) and when the low placental edge was more than 5 cm from the internal cervical os. Cesarean delivery for placental localization was indicated in women with complete or marginal placenta and performed (depending on clinical circumstances) when the placental edge was less than 2 cm from the internal cervical os in the third trimester. Cesarean delivery was planned at 37–38 weeks of gestation in stable women or earlier if severe bleeding occurred or if women went into labor [15].

### Outcomes

The primary outcome was prolongation of pregnancy defined by time interval in days from randomization to delivery. Maternal secondary outcomes were bleeding recurrence, need for blood transfusion, immediate pre-delivery hemoglobin count, cesarean delivery and indications, post delivery hemoglobin count, use of additional uterine devascularisation procedure or peripartum hysterectomy, length of hospitalization and death. Bleeding recurrence was defined as when clinical intervention was necessary i.e. tocolyis therapy or caesarean section due to hemorrhage before 37 + 0 weeks gestation. Placenta accreta was diagnosed according to clinical and histological criteria as previously reported [16]. Neonatal secondary outcomes were term of birth, birthweight, Apgar score less than 7 at 5 minutes, umbilical arterial pH < 7.00, hospitalization in neonatal intensive care unit, length of hospitalization, adverse neonatal outcomes associated with prematurity (only documented if premature birth occurred before 34 weeks of gestation) and perinatal death. Adverse perinatal outcome was defined as perinatal mortality and serious morbidity including chronic lung disease, neonatal sepsis, severe intraventricular hemorrhage greater than grade 2, periventricular leukomalacia greater than grade 1 and necrotizing enterocolitis [17].

### Sample size

No previous study has specifically evaluated the efficacy of maintenance oral nifedipine in pregnant women with preterm bleeding placenta previa. In a retrospective series published by Besinger et al. (1995), women treated with long-term maintenance tocolysis with oral or subcutaneous terbutaline had greater prolongation of pregnancy than those treated with short-term intravenous magnesium alone of more than three weeks (40.9 versus 15.3 days) [9]. In a randomized trial, [18] maintenance nifedipine therapy after discontinuation of acute intravenous tocolysis in women with preterm labor with intact membranes led to a 2 week increase in time elapsed between randomization and delivery when compared with no treatment (mean ± standard deviation 26.65 ± 18.89 vs. 16.14 ± 12.91 days). In view of these data, sample size calculations were based on a two-week difference and conservative standard deviation value of 25 days. In order to obtain 80% power for Student’s t-test at the two-sided 0.05 level, the target sample size was 104 women (52 per group). As potential loss to follow-up in each group was estimated at 5%, total sample size was set at 110 women.

### Randomization

Women were randomly assigned to receive nifedipine or placebo (allocation ratio 1:1). Group assignment was stratified by center and based on a computer generated random sequence in balanced blocks (Department of Biostatistics, Rouen, France). Treatment packages were prepared and numbered according to this random sequence and delivered by each local center pharmacy following each new inclusion. Treatment assignment and block size were blinded to investigators, participants, clinicians, and research nurses. Group assignments were also placed in sealed opaque sequentially numbered envelopes in association with a 24-hour telephone service for unblinding treatment allocation if necessary.

### Statistical analysis

In accordance with the intention-to-treat principle, all randomised patients were included in the analysis even in cases of discontinuation of treatment. No interim analysis was planned or performed. Two arm treatment comparisons relied on Student’s t-test for continuous outcomes, Pearson’s chi-square test or Fisher’s exact test as appropriate for categorical outcomes, and the log rank test for time to delivery for which a hazard ratio was also estimated from Cox proportional hazard model using the placebo group as the reference. Two-sided p-values less than 0.05 were considered significant. For each dichotomous outcome, relative risk (RR) and associated 95% confidence interval (95% CI) were estimated from the corresponding 2x2 table. Continuous variables are presented as means with standard deviations, unless otherwise specified. Software SAS (SAS Institute, Cary, NC, Version 9.3) was used.

## Results

**Fig 1:**
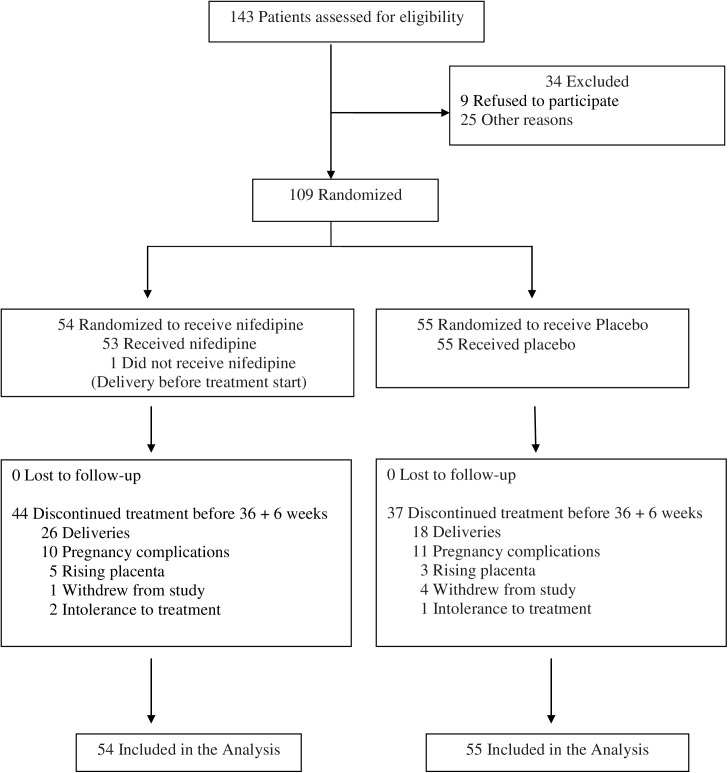
Flow chart of study participants.

A total of 143 potentially eligible women were approached during the study period, 9 of whom declined to participate in the trial. A total of 25 women were excluded for diverse reasons including initial suspicion of premature rupture of membranes though later unconfirmed (n = 14), persistent bleeding at 48 hours of acute tocolysis (n = 6), long term used for tocolytic therapy before the acute tocolysis protocol, (n = 2), uncertain date of conception (n = 1), maternal side effects reported during acute tocolysis (n = 2). Since one treatment batch could not be attributed due to blister expiration, a total of 109 women were enrolled in the trial (Fig 1). Of these, 54 women were assigned to the nifedipine group and 55 to the placebo group. One woman in the nifedipine group did not start treatment as she was delivered on randomization day due to severe bleeding and fetal distress. Treatment allocation was never unblinded during the study. The baseline characteristics of the randomized population were similar in the two groups (Table 1**)**. Most women were parous (69.8%) and 27.9% had undergone previous cesarean section. Distances from the placental edge to the internal cervical os at transvaginal sonography were as follow: overlaps (n = 65; 59.6%), between ≥ 0 mm to < 20 mm (n = 28; 25.7%), between ≥ 20 mm to < 35 mm (n = 16; 14.7%), and no woman between 35 mm to 50 mm; and were comparable between the two groups. Mean (Standard deviation: SD) gestational age at randomization was 29.2 weeks (2.5 weeks) in the nifedipine group and 29.4 weeks (2.6 weeks) in the placebo group. Acute tocolysis therapy were either nifedipine (n = 82; 75.2%), atosiban (n = 8; 7.3%), or twice consecutively (n = 18; 16.5%) and were comparable between the two groups.

**Table 1:** Baseline Demographics and Clinical Characteristics.

Characteristic	Nifedipine (n = 54)	Placebo (n = 55)
Maternal age, mean (SD), years	31.2 (5.9)	30.6 (4.6)
Maternal BMI, mean (SD), kg/m2	22.9 (3.5)	23.8 (5.1)
Nulliparous, No. (%)	16 (29.6)	17 (30.9)
Prior cesarean delivery, No. (%)	14 (25.9)	10 (18.2)
Prior placenta previa, No.n (%)	4 (7.4)	5 (9.0)
Prior abortion or curettage, No. (%)	14 (25.9)	21 (38.2)
Gestational age at study entry, mean (SD), weeks	29.2 (2.5)	29.4 (2.6)
Placental edge overlapping internal os, No. (%)	29 (53.7)	36 (65.4)
Anterior placenta previa, No. (%)	21 (38.9)	24 (43.6)
Placenta accreta, No. (%)	6 (11.2)	7 (12.9)
Initial systolic blood pressure, mean (SD), mmHg	113.9 (11.0)	115 (12.1)
Initial diastolic blood pressure, mean (SD), mmHg	66.7 (8.4)	69.2 (9.5)
Initial hemoglobin count, mean (SD), g/dL	11.2 (1.2)	11.3 (1.0)
Corticosteroids, No. (%)	47 (87.0)	46 (83.6)
Immediate transfusion, No.n (%)	1 (1.8)	1 (1.8)
Intravenous iron perfusion, No. (%)	4 (7.4)	3 (5.4)
Oral iron supplement, No.n (%)	50 (92.5)	51 (92.7)

BMI, body mass index; SD, Standard Deviation

### Primary outcome

**Fig 2:**
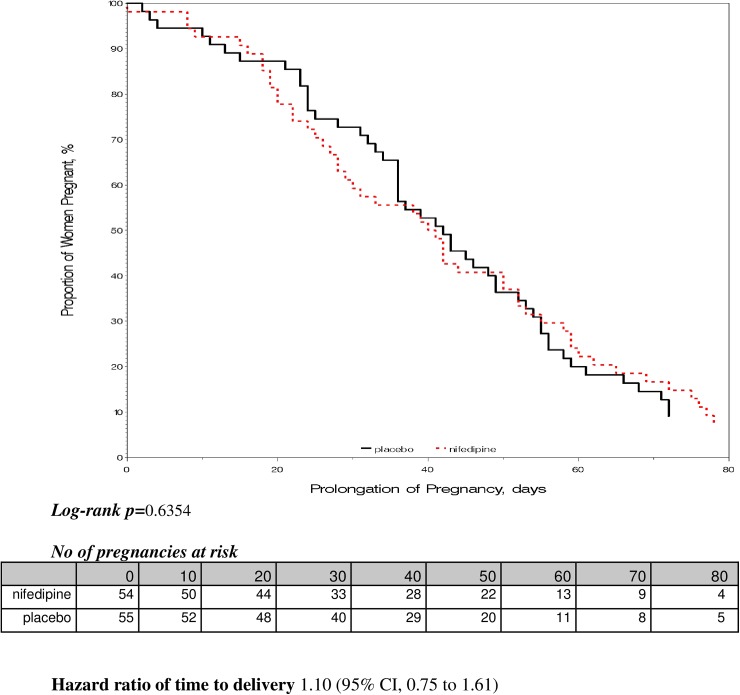
Prolongation of pregnancy after randomization.

Mean (SD) prolongation of pregnancy was not significantly different between the groups, 42.5 days (23.8 days) for the nifedipine group and 44.2 days (24.5 days) for the placebo group (p = 0.70, Student's t-test). Accordingly, distributions of time to delivery did not differ between groups (HR, 1.10; 95% CI, 0.75–1.61), and the Kaplan-Meier curve indicated no difference (log-rank *P*, .63; Fig 2). Mean (SD) gestational age at delivery was comparable between groups; 35.6 weeks (2.9 weeks) for the nifedipine group and 36.2 weeks (2.9 weeks) for the placebo group (p = 0.22, Student’s t-test; Table 2). Delivery prior to 37 weeks (plus 0 days) gestation occurred more frequently in the nifedpine group (36/54 women: 66.6%) in comparison with the placebo group (26/55 women: 47.2%) (RR, 1.41; 95% CI, 1.01–1.95; **Table 2**).

**Table 2:** Neonatal Outcomes.

Perinatal outcome		Nifedipine (n = 54)	Placebo (n = 55)	RR (95% CI)	P
Gestational age at birth, mean (SD), weeks		35.6 (2.9)	36.2 (2.9)		0.22
Delivery, No. (%)					
	< 32 weeks	8 (14.8)	7 (12.7)	1.16 (0.45–2.99)	0.75
	< 34 weeks	14 (25.9)	11 (20.0)	1.30 (0.64–2.60)	0.46
	< 37 weeks	36 (66.7)	26 (47.3)	1.41 (1.01–1.98)	0.04
Birth weight, mean (SD), grams		2568.3 (631.8)	2636.8 (712.0)		0.73
Apgar score at 5 min < 7, No. (%)		2 (3.7)	2 (3.6)	1.02 (0.15–6.97)	1.00
Umbilical arterial pH < 7.00, No. (%)		1 (1.9)	0 (0.0)		0.48
Hemoglobin at birth, mean (SD), g/dl		14.1 (2.6)	14.0 (2.1)		0.98
Neonatal Intensive Care Unit admission, No. (%)		23 (42.6)	18 (32.7)	1.30 (0.80–2.12)	0.29
Total hospital admission, mean (SD), days		15.8 (14.8)	15.1 (16.9)		0.46
Adverse perinatal outcome*, No. (%)		2 (3.8)	3 (5.5)	0.52 (0.10–2.61)	0.62
Perinatal death, No. (%)		0	0		

SD, Standard Deviation. CI, confidence interval. RR, relative risk (taking the placebo group as the reference).

*Adverse perinatal outcome was a composite of perinatal death, chronic lung disease, neonatal sepsis, intraventricular hemorrhage (IVH) greater than grade 2, periventricular leukomalacia (PVL) greater than grade 1 and necrotizing enterocolitis.

### Secondary outcomes

Neonatal outcomes were comparable between the two groups (Table 2). Adverse neonatal outcomes had similar frequency; 3.8% in the nifedipine group and 5.5% in the placebo group (RR, 0.52; 95% CI, 0.10–2.60, Table 2). No perinatal death was observed in either group.

Regarding maternal outcomes, at least one bleeding recurrence occurred in 37 out of 54 women (69%) in the nifedipine group and in 30 out of 55 women (55%) in the placebo group (RR, 1.26; 95% CI, 0.93–1.70, Table 3). Cesarean delivery rates were similar in the two groups; 74% in the nifedipine group versus 76% in the placebo group (RR, 0.97; 95% CI, 0.78–1.20). Cesarean due to hemorrhage performed before 37 weeks occurred more frequently in the nifedipine group in comparison with the placebo group (RR, 1.66; 95% CI, 1.05–2.72). A significantly higher number of women received post delivery intravenous iron perfusion in the nifedipine group in comparison with the placebo group: 29.6% versus 12.7% (RR, 2.32; 95% CI, 1.04–5.20). No maternal death was observed in either group.

**Table 3:** Maternal Outcomes.

Maternal outcome		Nifedipine (n = 54)	Placebo (n = 55)	RR (95% CI)	P
At least one bleeding recurrence, No. (%)		37 (68.5)	30 (54.5)	1.26 (0.93–1.70)	0.13
	with celestone rescue, No. (%)	6 (19.3)	3 (13.0)	1.63 (0.46–5.78)	0.44
At least 1 transfusion before delivery, No. (%)		0 (0.0)	1 (1.8)	0	1.00
At least 1 intravenous iron perfusion before delivery, No. (%)		2 (3.7)	3 (5.4)	0.68 (0.12–3.90)	1.00
Pre delivery hemoglobin, mean (SD), g/dl		11.1 (1.4)	11.4 (1.1)		0.30
Cesarean delivery, No. (%)		40 (74.1)	42 (76.4)	0.97 (0.78–1.20)	0.78
	Due to hemorrhage, No (%)	29 (72.5)	20 (47.6)	1.52 (1.05–2.20)	0.02
Cesarean due to hemorrhage					
	Occuring at any time, No. (%)	29 (53.7)	20 (36.4)	1.48 (0.96–2.27)	0.07
	Occuring < 37 weeks, No. (%)	26 (48.1)	16 (29.1)	1.66 (1.01–2.72)	0.04
Post delivery hemoglobin*, mean (SD), g/dl		9.5 (1.6)	10.1 (1.4)		0.11
Post delivery transfusion, No. (%)		9 (16.6)	8 (14.5)	1.15 (0.48–2.75)	0.76
Post delivery intravenous iron perfusion, No. (%)		16 (29.6)	7 (12.7)	2.33 (1.04–5.21)	0.03
Surgical hemostasis**, No. (%)		4 (7.4)	7 (12.7)	0.58 (0.18–1.87)	0.36
Length of stay in hospital, mean (SD), days		7.1 (3.9)	6.5 (3.5)		0.25
Maternal deaths, No. (%)		0	0		

SD, Standard Deviation. CI, confidence interval. RR, relative risk (taking the placebo group as the reference).

*Excluding women who received a blood transfusion during cesarean section

**Surgical hemostasis: cesarean-hysterectomy for placenta accreta (4 women in both groups), uterine arterial ligation (3 women in the placebo group)

Median (interquartile) overall compliance for treatment was respectively 0.94 (0.87–1.01) for the nifedipine group and 0.95 (0.89–1.00) for the placebo group. Maternal side effects were comparable between groups at one month of treatment; 7.4% (4/54) for the nifedipine group and 3.6% (2/55) for the placebo group (p = 0.66). Two women in the nifedipine group and one in the placebo group stopped therapy due to adverse effects.

### Sub-group analyses in women with placental edge below 20 mm from the internal os

Sub group analysis revealed a significantly lower mean gestational age at birth in the nifedipine group in comparison with the placebo group (35.4 weeks (2.8) versus 36.5 weeks (2.6)), with more frequent deliveries occurring before 37 weeks in the nifedipine group (RR 1.58 (1.10–2.27, Table 4). Regarding maternal outcomes, lower post delivery hemoglobin count (9.4 g/dL (1.4) versus 10.1 g/dL (1.4)) was reported in the nifedipine group. Moreover, at least one bleeding recurrence (RR 1.39 (1.00–1.93), and cesarean due to hemorrhage (RR 1.61 (1.03–2.52) were reported more frequently in the nifedipine group in comparison with the placebo group (Table 5).

**Table 4:** Neonatal Outcomes among women with placental distance of less than 20 mm.

Perinatal outcomes		Nifedipine (n = 47)	Placebo (n = 46)	RR (95% CI)	P
Gestational age at birth, mean (SD), weeks		35.4 (2.8)	36.5 (2.6)		0.04
Delivery, No. (%)					
	< 32 weeks	8 (17.0)	4 (8.6)	1.96 (0.63–6.06)	0.24
	< 34 weeks	12 (25.5)	8 (17.3)	1.47 (0.66–3.26)	0.34
	< 37 weeks	34 (72.3)	21 (45.6)	1.58 (1.10–2.27)	0.01
Birth weight, mean (SD), grams		2489.1 (616.7)	2663.0 (663.4)		0.29
Apgar score at 5 min < 7, No. (%)		2 (4.2)	2 (4.3)	0.98 (0.14–6.65)	1.00
Umbilical arterial pH < 7.00, No. (%)		1 (2.1)	0 (0.0)		1.00
Hemoglobin at birth, mean (SD), g/dl		13.8 (2.7)	13.9 (2.3)		0.62
Neonatal Intensive Care Unit admission, No. (%)		21 (44.6)	14 (30.4)	1.47 (0.86–2.52)	0.16
Total hospital admission, mean (SD), days		16.4 (14.5)	13.4 (13.5)		0.12
Adverse perinatal outcome*, No. (%)		2 (4.2)	2 (4.3)	0.67 (0.12–3.81)	1.00
Perinatal death, No. (%)		0 (0.0)	0 (0.0)		

SD, Standard Deviation CI, confidence interval; RR, relative risk (taking the placebo group as the reference)

*Adverse perinatal outcome was a composite of perinatal death, chronic lung disease, neonatal sepsis, intraventricular hemorrhage (IVH) greater than grade 2, periventricular leukomalacia (PVL) greater than grade 1 and necrotizing enterocolitis.

**Table 5:** Maternal Outcomes among women with placental distance of less than 20 mm.

Maternal outcomes		Nifedipine (n = 47)	Placebo (n = 46)	RR (95% CI)	P
At least one bleeding recurrence, No. (%)		34 (72.3)	24 (52.1)	1.39 (1.00–1.93)	0.05
	with celestone rescue, No. (%)	6 (20.6)	2 (11.1)	2.16 (0.49–9.50)	0.43
At least 1 transfusion before delivery, No. (%)		0 (0.0)	0 (0.0)		
At least 1 intravenous iron perfusion before delivery, No. (%)		2 (4.2)	2 (4.3)	0.98 (0.14–6.66)	0.55
Pre delivery hemoglobin, mean (SD), g/dl		11.1 (1.4)	11.5 (1.1)		0.16
Cesarean delivery		39 (82.9)	37 (80.4)	1.03 (0.85–1.25)	0.75
	Due to hemorrhage, No. (%)	28 (71.7)	17 (45.9)	1.56 (1.05–2.33)	0.02
Cesarean due to hemorrhage					
	Occuring at any time, No. (%)	28 (59.5)	17 (36.9)	1.61 (1.03–2.52)	0.03
	Occuring < 37 weeks, No. (%)	25 (53.1)	14 (30.4)	1.75 (1.05–2.92)	0.03
Post delivery hemoglobin*, mean (SD), g/dl		9.4 (1.4)	10.1 (1.4)		0.02
Post delivery transfusion, No. (%)		9 (19.1)	8 (17.3)	1.10 (0.47–2.61)	0.82
Post delivery intravenous iron perfusion, No. (%)		15 (31.9)	5 (10.8)	2.94 (1.16–7.42)	0.02
Surgical hemostasis**, No. (%)		4 (8.5)	6 (13.0)	0.65 (0.20–2.16)	0.48
Length of stay in hospital, mean (SD), days		7.3 (3.7)	6.5 (3.4)		0.07
Maternal deaths, No. (%)		0 (0.0)	0 (0.0)		

*Excluding women who received a blood transfusion during cesarean section

**Surgical hemostasis: cesarean-hysterectomy for placenta accreta (4 women in both groups), uterine arterial ligation (3 women in the placebo group)

## Discussion

### Main findings

Our randomized controlled trial in women with preterm bleeding placenta previa showed that maintenance of nifedipine therapy did not significantly prolong pregnancy when compared with placebo. However, our study also reported increased rates of cesarean due to hemorrhage in women delivered by cesarean section or when the surgical procedure was performed before 37 weeks in the nifedipine group as well as an increased rate of post delivery intravenous iron perfusion.

### Strengths and limitations

The strengths of our study include the significance of the clinical problem for both mother and neonate, the relatively large size of this randomized trial and the enrollment of women with high risk symptomatic placenta previa, 75% of whom were delivered by cesarean section. Our trial reports to our knowledge several maternal and neonatal outcomes associated with hemorrhage and prematurity in women with preterm bleeding placenta previa treated by prolonged tocolytics. Only a few women declined to participate in the study and compliance to treatment was excellent, with a very low rate of protocol treatment interrupted due to intolerance. We chose to treat women by nifedipine which is at present considered to provide the highest probability of delaying delivery, while improving neonatal and maternal outcomes in women at risk of preterm delivery [19].

Our trial has some limitations. Firstly we chose prolongation of pregnancy as the primary outcome although neonatal outcomes associated with prematurity may be considered of greater relevance. A recent large randomized controlled trial chose a composite adverse perinatal outcome as primary outcome to evaluate the efficacy of maintenance nifedipine in women with threatened preterm labor [17]. Despite recruitment of 406 women for the afore-mentioned trial, the authors failed to obtain adequate statistical power to enable further conclusions, in particular as the rate of adverse perinatal outcomes was lower than expected in the placebo group (13% instead of the expected 25% rate). We also report a very low rate of 5.5% of adverse perinatal outcome in the placebo group, bearing in mind that severe prematurity below 32 weeks was only observed in 14.4% of our overall population. An expected 44% relative reduction in adverse perinatal outcome, as in the trial published by Roos et al. [17] (reduction from 25% in the composite neonatal outcome in the placebo group to 14% in the nifedipine maintenance tocolysis group), would require randomising 2,242 women with preterm bleeding placenta praevia overall (reduction from 5.5% in the placebo group to 3.1% in the nifedipine group), which does not seem very realistic.

Secondly, we chose to treat women experiencing moderate recurrent bleeding with further acute tocolysis, so as to enhance the success of conservative management in the placebo group. In doing so, repeat acute tocolysis in the placebo group for recurrent bleeding may have minimized the observed effect of the maintenance of nifedipine treatment on prolongation of pregnancy.

### Interpretation

Only few studies published to date support prescribing a tocolytic in cases of preterm symptomatic placenta previa, as this treatment allows significant prolongation of pregnancy and the administration of a full course of corticosteroid for fetal maturation [9,10,11]. These studies suffer however from methodological problems and tocolytic treatments used lack proven tocolytic effects (magnesium sulfate) or incur adverse maternal and fetal side effects (beta-mimetics). The earliest study was retrospective comparing pregnancy outcomes following treatment with (75% of patients) or without tocolysis [10]. Women treated with long-term maintenance tocolysis with oral or subcutaneous terbutaline had an average of 40 days elapsing between admission and delivery which is comparable to our randomized groups. However, the authors also reported a trend for increasing risk of bleeding recurrence and need for transfusion in the tocolysis group. We also reported, in our sub-group of women with placental edge below 20 mm from the internal os, increased rates of recurrent bleeding and cesarean section due to hemorrhage in the nifedipine group. Only one published study was prospective and randomized but was based on a smaller sample [11]. This study was not blinded since some patients received a beta-mimetic intramuscularly for one week (10 mg of ritodrine every 6 hours) and others no treatment. The prolongation of pregnancy beyond 48 hours and overall length of pregnancy after starting ritodrine treatment were significantly increased compared to the control group without treatment. All these previous studies suffer from limited information on the maternal and neonatal morbidity with placenta previa and use of tocolytics.

Only data issued from few and small retrospective studies suggest a new classification using 20 mm placental edge distance from the internal os cut-off to better selected women with high risk cesarean delivery with hemorrhage [20]. However, poor information on the likelihood of antepartum hemorrhage with treatment decisions and based on placental edge distance is actually available. High rates of antepartum hemorrhage (28.2%) and emergency cesarean section (37%) have been reported in women with placental edge distance from 2.1 to 3.5 cm [21]. In fact, clinical outcomes in women with preterm bleeding placenta i.e. recurrent or heavy bleeding, emergency delivery for hemorrhage, and premature delivery could be highly variable and mostly irrespective of the degree of placenta [22]. Interestingly, few women with placental edge distance from 2.1 to 3.0 cm were included in our trial. In addition, placental migration rates are more frequently observed during the third trimester in women with low lying placenta in comparison with women with placenta overlapping or reaching the internal os [23]. We therefore included women with low lying placenta and also decided to prolong tocolytic therapy until 36 weeks + 6 so as to reduce recurrent bleeding events with significant placental migrations and therefore improve overall maternal outcomes. However, our finding cannot exclude a deleterious hemorrhagic impact related to nifedipine therapy in preterm bleeding placenta and particularly in women with placental edge below 20 mm from the internal os. Further studies are still needed to determine the best tocolytic agent women with preterm moderate bleeding placenta previa. An increased rate of late preterm neonates was observed in the nifedipine group and was most likely attributable to cesarean section due to hemorrhage and/or recurrent bleeding.

## Conclusion

Our results do not support the use of maintenance nifedipine therapy in women with preterm bleeding placenta previa owing to a lack of clinical significance concerning neonatal benefits and to an increase in cesarean delivery rate due to hemorrhage.

## Supporting information

S1 File CONSORT 2010 Checklist. (DOC)

S2 File PROTOCOL. (DOC)

S3 File 170105DataSetPlosOne. (XLSX)
