# Supplementary material for: Maintenance nifedipine therapy for preterm symptomatic placenta previa: A randomized, multicenter, double-blind, placebo-controlled trial
Source: PLoS One. 2017 Mar 23;12(3):e0173717. doi: 10.1371/journal.pone.0173717 (PMC5363821; doi:10.1371/journal.pone.0173717)
Supplement: S2 File — (DOC) [file pone.0173717.s002.doc]

I. Introduction and rationale for the study

I.1 Definition of the disease or event studied

Placenta previa (PP) is a complication occurring in approximately 5% of full-term pregnancies and is defined as a placenta inserted along all or part of the lower segment. This abnormal location of the placenta can cause severe and repeated bleeding, indicating the gravity of this pathology. Thus, historically in the absence of treatment or appropriate patient care, spontaneous maternal and fetal mortality attained levels of around 25% and 90% respectively. Advances in resuscitation techniques and in particular in caesarean section have led to significant improvements in PP prognosis. There remains however, a high risk of complications linked to hemorrhage in the mother and induced prematurity in the infant.

I.2 Current scientific knowledge

Over the last twenty years, treatment of placenta previa bleeding has readily become conservative, providing a prolongation of pregnancy which allows a potential gain in maturity in the fetus. Conservative treatment can only be envisaged preterm and when bleeding remains moderate, i.e. with no immediate threat to life. This treatment includes constant monitoring in hospital associated with initial tocolysis for at least 48 hours. Tocolysis aims to establish fetal maturation by way of corticosteroids (Célèstène chronodose® 12 mg in a single intramuscular injection, 2 consecutive days), but also to decrease the contractile activity of the uterus that can cause bleeding. The reduction in contractile activity by a tocolytic agent is thus likely to stop or reduce the amount of bleeding thereby allowing pregnancy to continue. This beneficial effect of tocolysis is only reported in a small number of studies:

1- The earliest study, that of Bessinger et al. included a total of 112 cases of preterm placenta previa bleeding (1). This was a retrospective study comparing pregnancy outcomes following treatment with or without tocolysis. Tocolytic treatment prescribed in this study (75% of patients) varied considerably depending on the type of agent used (most frequently magnesium sulfate or beta-mimetics) and how it was administered (treatment route, duration, dosage), reflecting the significant variations in methods. Patients with tocolysis had a duration of pregnancy (39. 2 +/- 24.3 days versus 26.9 +/- 23.8 days, p = 0.03) and newborns with birth weight (2520 +/- 2124 +/- 755 grams versus 965 grams, p = 0.02) which were significantly increased compared to patients without tocolysis. The rates for transfusion, recurrent bleeding and neonatal outcomes were similar in both groups. Side effects due to tocolytic treatments were not reported in this study.

2- The study by Towers et al. was also retrospective and included both placenta previa (n = 105) and retroplacental hematomas (n = 131) (2). Both the methodology and reported results reflect those of Besinger et al. Treatment duration averaged 16 days, with a median treatment of 13 days.

3- The most recent published study was prospective and randomized but was based on a smaller sample (n = 60) (3). This study was not blinded since some patients (n = 30 patients) received a beta-mimetic intramuscularly for one week (10 mg of ritodrine every 6 hours) and others (n = 30 patients) no treatment. The prolongation of pregnancy beyond 48 hours (93.3% versus 50%, p <0.05) and overall length of pregnancy (25.3 +/- 17.7 days versus 14.47 +/- 20.3 days, p <0.05) after starting ritodrine treatment were significantly increased compared to the control group without treatment. Hemoglobin count was significantly higher in the treated group after one week of treatment compared to the control group (10.4 g / 100 ml +/- 1.3 versus 9.3 g / 100 ml +/- 1.2, p <0.01). The frequency of recurrent bleeding was lower in the treated group compared to the untreated group though not significantly (66.6% versus 76.6%). No neonatal outcomes or side effects linked to treatment were reported.

**These few studies published to date support prescribing a tocolytic in cases of hemorrhagic PP, as this treatment allows significant prolongation of pregnancy and the administration of a full course (48 hours) of corticosteroid for fetal maturation (1,2,3).** These studies suffer however from methodological problems and are inadapted to our clinical practice. In fact the tocolytic treatments used in these studies lack proven tocolytic effects (magnesium sulfate), incur adverse maternal and fetal side effects (beta-mimetics) or are aggressively administered (repeated administration of beta-agonists intramuscularly).

In November 2002 the French National College of Gynecologists and Obstetricians brought out guidelines for clinical practice, concerning the threat of preterm labor with intact membranes (4). **Three classes of first-line tocolytics were included in these recommendations: beta-mimetics, calcium channel blockers and oxytocin antagonists.** Short-term tocolysis over 48 hours was recommended as it provides time to complete corticosteroid treatment for foetal growth. The guidelines also state that tolerance of calcium channel blockers and oxytocin antagonists is superior to that of beta-mimetics.

The Cochrane Database published a meta-analysis of randomized controlled trials comparing the efficacy and tolerance of calcium channel blockers and beta-mimetics (a total of twelve randomized controlled trials with a total of 1029 patients) (5). This meta-analysis revealed better tocolytic efficacy of calcium channel blockers which significantly reduce the number of premature deliveries at one week (RR = 0.76 confidence interval at 95%; 0.6 to 0.97) and before 34 WA (RR= 0.83, 95% confidence interval; 0.69 to 0.99). Calcium channel blockers also significantly reduce early treatment interruption due to maternal intolerance (RR = 0.14, 95% confidence interval; 0.05 to 0.36), the frequency of newborn respiratory distress (RR = 0, 63-95% confidence interval from 0.46 to 0.88), the frequency of neonatal enterocolitis (RR = 0.21, 95% confidence interval; 0.05 to 0.96) and the frequency of newborn intraventricular hemorrhage (RR = 0.59, 95% confidence interval; 0.36 to 0.98) compared with beta-agonists. **The Cochrane Database concludes by recommending using calcium channel blockers for tocolysis**. The efficacy of oxytocin antagonists is equivalent to that of beta mimetics but with fewer adverse effects (6). There is however no reported improvement in neonatal prognosis after the administration of oxytocin antagonists. No study as yet has compared the efficacy and tolerance of calcium channel blockers and oxytocin antagonists. The cost of oxytocin antagonists is 100 times more expensive than for calcium channel blockers, the former being suitable only for parental administration over a period of 48 hours.

In practice, most teams are currently using first-line calcium channel blockers for tocolysis despite their unlicensed indication. Calcium channel blockers are also widely prescribed in cases of hemorrhagic PP despite there being no research of consequence on the subject.

I.3 Research hypothesis

No study exists in the field of hemorrhagic PP to determine whether prolongation of the duration of tocolysis (beyond 48 hours) compared to short tocolysis of 48 hours is beneficial to mother and child. All the above cited published studies compare the results of more or less extended tocolysis with no tocolysis.

Extended tocolysis compared to short tocolysis is likely to lead to permanent relaxing of the uterus, thus reducing the frequency and severity of bleeding, bringing possible improvements in maternal and fetal prognosis.

Calcium channel blockers may be considered first-line in long term tocolysis as they have few side effects, are easily taken by oral adminstration and have a low purchase price.

**In half of the studies in the Cochrane Database meta-analysis, tocolysis with nifedipine was continued for a period until at least 34 WA with a dosage of three 20 mg capsules daily.** One discontinuation of treatment was reported for maternal intolerance out of all the trials included in the meta-analysis (419 patients). Neonatal morbidity was significantly reduced following tocolysis with nifedipine (see above details) (5).

Other data exists from hypertensive pregnant patients receiving sustained treatment by channel blockers. A large randomized study (n = 154 patients with nifedipine versus no treatment n = 138 patients) evaluated the possible beneficial effects of administering long term nifedipine (daily doses between 20 and 80 mg), prescribed no earlier than 12 WA and no later than 34 WA in women with moderate, mostly chronic hypertension (7). No benefits were reported, nor were any maternal and neonatal complications associated with the extended administration of nifedipine. Rates of fetal loss, newborn birth weight, term at birth and the percentage of newborns hospitalized in intensive care units were comparable between the two groups.

I.4 The Risk/Benefit Ratio

The expected benefits for patients receiving extended tocolysis with nifedipine are of interest to both mother and child. Extended tocolysis is likely to reduce the frequency and severity of bleeding episodes and therefore limit maternal and fetal blood loss. The reduction in bleeding episodes allows a prolongation of pregnancy which is beneficial for mother and child. Prolonging pregnancy allows for gains in maturity and weight in the fetus and allows the mother to have a more mature newborn, more often able to stay with her. This prolongation also allows some patients to give birth by natural means at a later date. Indeed some placenta previa may move away from the cervix as pregnancy progresses and no longer be previa.

Patients not receiving extended tocolysis will benefit from current, standard care given for this type of pathology. Tocolysis can be renewed in cases of rebleeding.

The expected risks would be associated with a prolonged administration of nifedipine, as all other patient care follows current standard practice. Adverse effects of calcium channel blockers are usually very mild and only rarely require stopping treatment. These adverse effects usually occur at the start of treatment, essentially in the form of headaches and transient flushing. Arterial hypotension in non-hypertensive pregnant women is rare. The Cochrane Database meta-analysis report no cases of symptomatic hypotension warranting cessation of treatment (5). The expected significant risk to the mother may be the appearance of an allergic reaction justifying an immediate interruption of treatment and leaving the protocol.

A fetotoxic effect of calcium channel blockers has been reported in animals. This effect has never been reported in humans (Vidal dictionary). The Cochrane Database meta-analysis reports no cause to suggest possible toxicity linked to calcium channel blockers. The analysis however reports lower neonatal morbidity when using calcium channel blockers compared to beta-agonists.

II. Study Objectives

The objective of this study is to determine whether extended tocolysis with a calcium inhibitor may improve maternal or fetal prognosis in cases of low-lying hemorrhagic placenta.

II.1 Main objective

The main objective of this study is to assess the benefits of extended tocolysis with a calcium inhibitor on the duration of prolongation of pregnancy from the initial bleeding episode.

II.2 Secondary Objectives

- To evaluate the side effects of nifedipine treatment in cases of extended tocolysis.

- To evaluate a possible reduction in maternal blood loss in cases of extended tocolysis while assessing the need or not for transfusion or interventional hemostasis techniques.

- To evaluate possible improvement in neonatal prognosis in cases of extended tocolysis while assessing term at birth, birth weight and immediate neonatal morbidity.

III. Study Design

III.1 Methodology

This is a prospective, therapeutic, multicentered (5 centers), double-blinded randomized, study. Any patient pregnant for a minimum of 24 WK, hospitalized for bleeding placenta previa will initially receive short-term tocolysis by calcium inhibitor or oxytocin inhibitor for 48 hours in order to establish maturation by corticosteroids. After the 48 hours, patients wishing to participate in the study will be randomized.

Patients in group A (control group) will receive a placebo, while patients in group B will receive extended tocolysis with a calcium channel blocker until completion of 37 WK.

III.2 Provisional schedule, duration of the trial

Estimated trial startdate: 2^nd^ to 3^rd^ trimester 2008.

Authorized period of patient enrollment: 24 months

Duration of trial participation for each patient: 4 months

Total trial duration 28 months (24 months + 4 months of inclusion (treatment + follow-up)

III.3 Study Population

III.3.1 Number of patients to be included

To highlight a difference in mean length of pregnancy prolongation, of 14 days between the two groups (15 days prolongation on average in the short tocolysis group versus 40 days in the extended tocolysis group, with different tocolytics used in Bessinger’s study) with a standard deviation of 25 days, it is necessary to have 52 subjects per group with a power of 80% and a p-value of 5% (bilateral formulation). The predicted number of patients lost to follow-up is low for this disease due to a necessity for regular medical monitoring in a designated maternity unit. Consequently and on the basis of a 5% loss to follow-up in each group, **the number of patients required for this study is 110**. **55 patients will therefore be included per group**.

Recruitment will be conducted in 5 centers.

III.3.2 Criteria for inclusion and non-inclusion

Patients pregnant for a minimum of 24 WK and hospitalized for bleeding placenta previa will be eligible to participate in this study. Patients who satisfy inclusion / non-inclusion criteria are to receive their randomized six- week treatment during the INCL inclusion visit (J 0).

**Inclusion criteria**

1. Low-lying placenta confirmed by transvaginal ultrasound, defined by a distance of less than 5cm between the lower edge of the placenta and the internal os.

2. Low-lying symptomatic placenta as defined by the occurrence of at least one bleeding episode.

3. Time of occurrence of the first episode of bleeding no earlier than 24 +0 weeks gestation and no later than 34 weeks gestation

4. Singleton pregnancy.

5. Age ≥ 18 years old.

6. A patient who has given free and informed consent and has signed the consent form.

7. Patient affiliated to a social security scheme.

**Criteria for non-inclusion**

1. Premature rupture of membranes.

2. Extensive initial bleeding justifying a rapid termination of pregnancy.

3. Unstable hemodynamics.

4. Abnormal fetal heart rate justifying a rapid termination of pregnancy.

5. Fetal death in utero, severe IUGR < 3rd percentile.

6. Associated maternal pathologies: pre-eclampsia, chorioamnionitis, cholestasis of pregnancy, hepatic steatosis and hepatocellular deficiency.

7. Suspected abruptio placentae.

8. Initial tocolysis < 48 hours.

9. Hypersensitivity to nifedipine, coronary heart disease

10. Medication taken: enzyme-inducing anticonvulsants, baclofen, cimetidine, itraconazole, phenytoin, rifampicin, alpha blockers, amifostine, tricyclic antidepressants, neuroleptics, beta-blockers.

III.4 Treatments

III.4.1 Description of treatment tested (legal notices in Appendix 2)

Nifedipine (Adalate LP 20 mg®, Laboratoire Bayer Pharma SAS) is a calcium channel blocker which obtained marketing authorization (MA in 1984, revised 2003) for high blood pressure.

The product is in the form of sustained release tablets (orange/pink) for oral administration of 20 mg nifedipine.

- These products are labeled in accordance with Article R.5123 of the Code of Public Health and with Appendix 13 recommendations in european Good Manufacturing Practices (GMP).

- These products will be distributed to various pharmacies in care facilities in accordance with Good Distribution Practices (GDP).

III.4.2 Randomization and Double Blind testing

**Randomization**

Randomization is carried out at the INCL inclusion visit, after verification of inclusion / non-inclusion criteria. Treatments are randomized with a 1:1 allocation for both treatments. Randomization will be stratified by center (n = 5) and the draw will be made in chronological order of arrival of patients, from randomization lists drawn up by the Rouen University Hospital Biostatistics Service (Pr. Jacques Benichou). These lists provide the randomization codes corresponding to treatment batch numbers.

These randomization lists will subsequently be forwarded to the service provider (Company LC2) who will prepare sealed envelopes, box therapeutic units, and produce unblinding envelopes for each center.

At each inclusion a processing number will be assigned to each patient. The treatment can then be issued by the central pharmacy at each center so that treatment can commence the following day.

**Double blind**

The treatment period in parallel groups is double-blinded. The blind will be maintained until final data analysis.

The treatment code (type of treatment) assigned to each patient will be kept by the sponsor, by the main investigator and by the central pharmacy at each center in sealed envelopes.

In cases where unblinding is needed, i.e. in the event of severe adverse events requiring knowledge of the assigned treatment for patient care, the investigator should first try to contact the trial coordinating investigator before disclosure of the treatment code.

If an envelope is opened, the reason, the opening date and the signature of the investigator should be noted on it. At the end of the study, during the final visit, all envelopes (sealed and opened) will be collected and returned to the promoter.

III.4.3 Tracking information and study products

Treatment distribution will be carried out by the Company LC2, Packaging and Logistics Laboratory for clinical batches (Lentilly 69210).

The numbered batches will be distributed to different centers. Each batch will initially include six weeks of treatment to minimize costs. Should this treatment time prove insufficient, another batch will be given to the patient. Each patient will receive a placebo, or the Adalate LP 20 mg ® in tablet form.

III.4.4 Treatment procedure

All patients will initially be hospitalized for at least 48 hours to monitor the extent of bleeding and assess hemodynamic status (normal standard blood test in this context includes CBC, PT, APTT, antiglobulin testing, blood pressure monitoring every 6 hours).

All patients initially receive tocolysis with nifedipine or tractocile for 48 hours: 1 capsule of nifedipine (Adalate®) 10 mg by oral route renewable every 20 minutes up to 4 capsules per hour, followed by long-acting nifedipine for 48 hours (Adalate LP 20mg®, one 20 mg tablet, 3 times daily) or tractotile (Atosiban®).

All patients initially receive an injection of Célèstène chronodose® 12 mg in a single intramuscular injection, 2 consecutive days.

All patients will receive preventive iron therapy: 80mg Tardyferon ®, 2 tablets and 5mg Spéciafoldine ®, 1 tablet daily.

**The following treatment protocol** will then be offered to patients after the first 48 hours of hospitalization. The protocol will commence only after validation of inclusion / non-inclusion criteria and reception of patient consent.

After randomization, a treatment batch will be given to the patient. Each batch will initially include treatment for six weeks to minimise costs. Should this length of time prove insufficient, another batch will then given to the patient. Depending on randomization, tablets for a given patient will be:

- Group A = Placebo

- Group B = Adalate LP 20mg ®

All patients will take 3 tablets daily, 1 tablet morning, noon and night.

The treatment will be continued without interruption until 36 weeks + 6 days.

Patients will be able to return home after the first bleeding episode if there is complete resolution of the episode and if the place of residence is at a distance of less than 30 minutes by car.

In case of rebleeding or the onset of painful contractions, further tocolysis by nifedipine or tractocile can be administered for 48 hours (in compliance with the initial tocolysis previously described). Tablets taken as part of the long- term treatment will be interrupted for the duration of this tocolysis. A second and final return home is possible under the same conditions as defined above.

In order to assess evolution in location of the placenta, monitoring by endovaginal ultrasound will be performed on a monthly basis.

Clinical monitoring will be carried out bi monthly to pinpoint possible intolerance to treatment or adverse effects.

III.4.5 Prohibited concurrent therapies

Enzyme-inducing anticonvulsants, baclofen, cimetidine, itraconazole, phenytoin, rifampicin, alpha blockers, amifostine, tricyclic antidepressants, neuroleptics, beta-blockers.

III.5 Visits et Evaluation

III 5.1 Trial procedure

Group 1: Placebo

Group 2: Adalate LP 20mg ®

Treatment by Placebo or Adalate LP 20mg ®

Fortnightly monitoring

Early discharge possible

Jours – Days

Visites – Visits

SEL – Screening visit

INCL – Inclusion visit

N – Maximum period for prescription of treatment

SOR: Discharge of patient and new-born infant from the maternity unit

III.5.2 Schedule of Visits

| **Tests** | **Initial Tocolysis** | **Randomization** | **Monitoring** | **Monitoring** | **Early discharge** | **Treatment interrupted** | **End** |
| --- | --- | --- | --- | --- | --- | --- | --- |
| Days | -1 | 0 | / 15 | / 30 | N - | N | N + |
| Visits | SEL | INCL | SURV 15 | SURV 30 | PREM | 36 WA + 6 | SOR |
| Patient information  Patient consent | X |  |  |  |  |  |  |
| Inclusion criteria  Non-inclusion criteria | X | X |  |  |  |  |  |
| Demographic data | X |  |  |  |  |  |  |
| Medical antecedents  Surgical antecedents |  | X |  |  |  |  |  |
| Full pathology history |  | X |  |  |  |  |  |
| Dispensing of treatment |  | X |  |  |  |  |  |
| Patient follow-up schedule |  | X |  |  |  |  |  |
| Patient blood pressure | X | X | X | X | X | X | X |
| Patient blood tests :  CBC / PT / APTT / antiglobulin | X |  |  |  |  |  | X |
| Endovaginal echography |  |  |  | X |  |  |  |
| Adherence to treatment |  |  | X | X | X | X | X |
| Overall tolerance to treatment  Satisfaction with treatment |  |  | X | X | X | X | X |
| Maternal data at trial completion  (See page 13 : Final visit) |  |  |  |  |  |  | X |
| Neonatal data at trial completion  (See page 13 : Final visit) |  |  |  |  |  |  | X |
| Effets / undesirable events |  | X | X | X | X | X | X |
| Concurrent treatment | X | X | X | X | X | X | X |

11

III.5.3. Visits schedule

**Screening visit : SEL visit (J-1)**

Evaluations are presented in Table III.5.2.

1. Verification of inclusion / non-inclusion criteria.

2. Obtain informed consent form signed by the patient.

3. Obtain history of the disease, past major medical and surgical history, and associated treatments.

**Inclusion Visit / Randomization and Start of treatment: Visit INCL (J 0)**

Randomization is performed after checking of inclusion / non-inclusion criteria. Treatments are randomized with a 1:1 allocation for both treatments.

Randomized patients will take their first tablet, Placebo or Adalate LP 20mg ®, the day after the INCL visit (J 0).

1. Verification of inclusion / non-inclusion criteria.

2. Take in the informed consent form signed by the patient.

3. Randomization.

4. Allocate patient treatment.

5. Plan a monitoring schedule with the patient.

6. Go through the adverse effects.

**Intermediate monitoring visits**

Evaluations as presented in Table III.5.2.

1. Look for possible allergy, flushing, symptomatic hypotension, headaches.

2. Blood pressure monitoring.

**Re- hospitalization**

In cases of bleeding recurrence

In cases of adverse effects.

**Treatment discontinuation no later than 36 weeks + 6 days**

Evaluations as presented in Table III.5.2.

**Final visit before discharge of mother and child from maternity unit**

For the mother:

1. blood count

2. treatment satisfaction

3. transfusion or prescribing of Venofer

4. Data collection concerning delivery and complications linked to immediate postpartum period.

For the child:

1. blood sample from cord at birth (placenta)

2. term at birth

3. birth weight,

4. length of hospitalization,

5. neonatal morbidity.

III.5.4 Assessment of effectiveness of treatment

**Primary efficacy parameters**

• **Significant prolongation of pregnancy**: time elapsed from the start of treatment to delivery are compared in each group.

**Secondary efficacy parameters**

• **Improved maternal prognosis:**  the two groups are compared as follows;

- Delta hemoglobin level at inclusion and in the immediate postpartum period,

- The need for transfusion or prescription of Venofer.

• **Improved neonatal prognosis:** the two groups are compared as follows;

- Birth weight,

- Term at birth,

- length of hospitalization,

- Morbidity, mortality.

III.5.5 Assessment of tolerance

**Assessment is carried out through questioning and by taking patient blood pressure at the various visits planned in the protocol.**

III.5.6 Assessment of compliance

Adherence to the study will be assessed by:

- Questioning of the patient at each follow-up visit,

- A count of the treatment units remaining and used (empty packaging) brought by the patient and relating to the 15 day period leading up to each follow-up visit.

III.6 Endpoints

III.6.1 Primary endpoint

The primary endpoint will be the duration in days of the prolongation of pregnancy from the initial bleeding episode.

III.6.2 Secondary Endpoints

Assessment criteria for secondary maternal endpoints will be:

- pregnancy prolongation beyond 15 days following the initial bleed,

- frequency of rebleeding,

- side effects of treatments,

- need for transfusion or the prescription of Venofer,

- emergency and hemorrhagic caesarean section,

- hemoglobin levels immediately before birth and in the immediate postpartum period,

- need for a hemostatic hysterectomy or embolization,

- Postpartum hospital stay,

- Maternal mortality.

Assessment criteria for neonatal secondary outcomes will be:

- birth weight,

- Apgar score at 5 minutes,

- arterial pH at birth,

- hemoglobin count taken from newborn cord

- hospitalization in intensive care unit

- length of hospitalization,

- Neonatal morbidity associated with prematurity

- Neonatal and perinatal mortality.

IV. Trial Drop-out

The following circumstances will be considered as sufficient to opt for early discontinuation:

1. Likely intolerance to treatment: e.g. persistent headache, flushing, maternal hypotension.

2. Discontinuation of treatment.

3. Occurrence of heavy bleeding or abnormal fetal heart rate justifying rapid termination of pregnancy.

4. Premature rupture of membranes during treatment.

5. In cases where additional maternal pathology justify a termination of pregnancy.

6. If ultrasound monitoring reveals a distance between the lower part of the placenta and the internal cervical os greater than 5 cm.

Any patient leaving the study due to an adverse event should be followed up until resolution of the adverse event.

In cases of early drop-out from the trial, the final observation report forms should be completed.

V. Statistical methods

V.1 Calculation of patient numbers

The patient number calculation is based on the duration of the prolongation of pregnancy from the initial bleeding episode (primary endpoint). The average length of prolongation of pregnancy and standard deviations used are derived from data published by Bessinger RE et al. To highlight a difference in mean length of prolongation of pregnancy of 14 days between the two groups (15 days average prolongation in the short tocolysis group versus 40 days in the extended tocolysis group) with a standard deviation of 25 days, 52 subjects are required per group with a power of 80% and a p-value of 5% (bilateral formulation). This number also allows at a power of 80% to detect a 26.5% difference in percentage of women for whom the prolongation of pregnancy will be longer than 14 days (50% for short tocolysis versus 76.5% for extended tocolysis).

V.2 Statistical Analysis

Comparisons of the two groups will be based on intention to treat analysis. For the primary endpoint, both groups will first be compared by means of the Student t-test and secondly by a linear regression model taking into account potential prognostic factors (type of placenta previa and gestational age at inclusion). The same type of analysis will be carried out for other quantitative variables. For binary criteria, the comparison between the two groups will be made using the Chi 2 test or the Fisher exact test if necessary, complemented by a logistic regression test to take into account other prognostic factors.

V.3 Feasibility Study

Four of the participating centers are level 4 maternity hospitals with neonatal intensive care units and one is a level 2B maternity hospital with a neonatology department. These centers, due to their structure, are able to manage placenta previa bleeding in clinical practice. The total number patients treated for placenta previa or low-lying placenta is on average 20 per year at Rouen University Hospital, half of the total patient numbers (see below).

The duration of the trial shall be 2 years.

VI. Bibliography

1/ Bessinger RE, Moniak CW, Paskiewicz LS, Fisher SG, Tomich PG. The effect of tolytic use in the management of symptomatic placenta previa. Am J Obstet Gynecol 1995; 172(6): 1770-8.

2/ Towers CV, Pircon RA, Heppard M. Is tocolysis safe in the management of third-trimester bleeding ? Am J Obstet Gynecol 1999; 180(6); 1572-8.

3/ Sharma A, Suri V, Gupta I. Tocolytic therapy in conservative management of symptomatic placenta praevia. Int J Gynecol Obstet 2004; 84: 109-13.

4/ Recommandations pour la pratique clinique. La menace d'accouchement prématuré à membranes intactes. J Gynecol Biol Reprod Nov.2002; 31(S7).

5/ King JF, Flenady VJ, Paptsonis DNM, Dekker GA, Carbonne B. Calcium channel blockers for inhibiting preterm labor. Cochrane Database of Systematic Reviews 2004.

6/ The worldwide Atosiban versus Beta-agonists Study Group. The effectiveness and safety of the oxytocin antagonist atosiban versus beta-adrenergic agonists in the treatment of preterm labor. Br. J Obstet Gynaecol 2001; 108:133-42.

7/ Gruppo di Studio Ipertensione in Gravidanza. Nifedipine versus expectant management in mild to moderate hypertension in pregnancy. Br J Obstet Gynaecol 1998; 105: 718-22.

8/ Carr DB, Clark AL, Kernek K, Spinnato JA. Maintenance oral nifedipine for preterm labor : a randomized clinical trial. Am J Obstet Gynecol 1999; 181(4): 822-7.
